# Evaluation of anterior translation in total knee arthroplasty utilizing stress radiographs

**DOI:** 10.1186/s13018-023-03862-x

**Published:** 2023-06-01

**Authors:** Sean P. Ryan, Niall H. Cochrane, William A. Jiranek, Thorsten M. Seyler, Samuel S. Wellman, Michael P. Bolognesi

**Affiliations:** Duke University, Durham, USA

**Keywords:** Flexion instability, Total knee arthroplasty, Stress radiographs, Anterior drawer, Telos

## Abstract

**Background:**

Flexion instability is a common cause for revision after total knee arthroplasty (TKA); however, little objective criteria exist to determine excessive laxity in flexion. This study sought to determine the reliability of stress radiographs for flexion laxity using manual stress as well as a commercially available flexion stress device, with the hypothesis that a commercially available force device would provide increased translation compared to manual stress, and radiographic measurements would be reproducible.

**Methods:**

Ten patients who previously underwent TKA with non-hinged components were prospectively and consecutively enrolled at a single center to undergo stress radiographs. Three lateral radiographs with the knee at 90° of flexion were obtained for each patient: rest, commercial stress device at 150N, and manual stress. Calibrated radiographs were evaluated by two raters, and inter-rater and intra-rater reliability were determined using intraclass correlation coefficients (ICC).

**Results:**

Ten patients (seven female) with mean age 72 (range 55–82) years and average duration from surgery 36 (range 12–96) months were evaluated. The commercial stress device provided significantly less anterior translation than manual stress (− 0.3 mm vs. 3.9 mm; *p* < 0.01). Two patients reported pain with use of the stress device. Inter-observer reliability of measurements was good for commercial stress (ICC = 0.86) and excellent for manual stress (ICC = 0.94). Eighty-five percent of measurements were within 1 mm between observers. Intra-observer reliability of measurements was good to excellent for both the stress device and manual stress.

**Conclusions:**

Lateral stress radiographs may assist in the objective evaluation of flexion instability. A commercially available product provided less translation than manual stress; however, measurements were reliable and reproducible between observers. Further research is required to correlate translation with stress radiographs to patient outcomes following revision arthroplasty.

## Introduction

Flexion instability is one of the three most common modes of failure of total knee arthroplasty (TKA) [1, 2]. While it was previously felt to be a problem only in patients with cruciate retaining (CR) knee designs, it is now recognized in posterior stabilized (PS) systems as well [3]. Patients with symptomatic flexion instability may report sensations of instability, recurrent effusions, persistent bursitis, and pain with loading the knee in flexion, particularly during stairs [4]. Unfortunately, despite its contribution to dissatisfaction after TKA, little data exist to guide the diagnosis or treatment.

To date, identifying flexion instability is subjective and surgeon dependent. Current diagnostic criteria include radiographic evaluation and physical examination. Lateral radiographs can be scrutinized to assess posterior condylar offset and posterior tibial slope, but no set parameters exist to guide this evaluation [5]. For this reason, physical examination remains the primary mode of diagnosis, with excessive (> 1 cm by convention) anterior–posterior (A–P) translation when the knee is flexed to 90° of flexion when performing anterior drawer [6]. However, the interpretation of A–P translation is surgeon dependent and no objective parameters exist, making it difficult to evaluate with physical exam alone [4]. The lack of quantitative guidelines for diagnosis of flexion laxity/instability likely contributes to worse outcomes for revision surgery when compared to revision TKA for other reasons [7, 8]. Without objective and reproducible diagnostic criteria, revision procedures are likely to continue to have varying degrees of success.

Unlike in other areas of orthopedics where stress radiographs have proven to be useful, this technique has been historically underutilized in adult reconstruction. While a KT-1000 has been used to evaluate native knees at low degrees of flexion in sports medicine [9], the literature is lacking regarding the evaluation of flexion laxity at 90° of flexion. Recently, a commercially available device has been utilized to evaluate A–P translation at 90° of flexion (Telos stress device; Austin & Associate, Fallston, Md.)[10, 11]. Thus, we sought to (1) compare translation with 150N set force with the Telos stress device to manual stress with lateral radiographs, and (2) to compare inter- and intra-rater reliability of calibrated radiographic measurements for tibial translation. The authors hypothesized that there would be comparable translation of the tibia between the Telos device and manual stress, and that there would be acceptable inter- and intra-rater reliability of translation measurements.

## Materials and methods

**Fig. 1:**
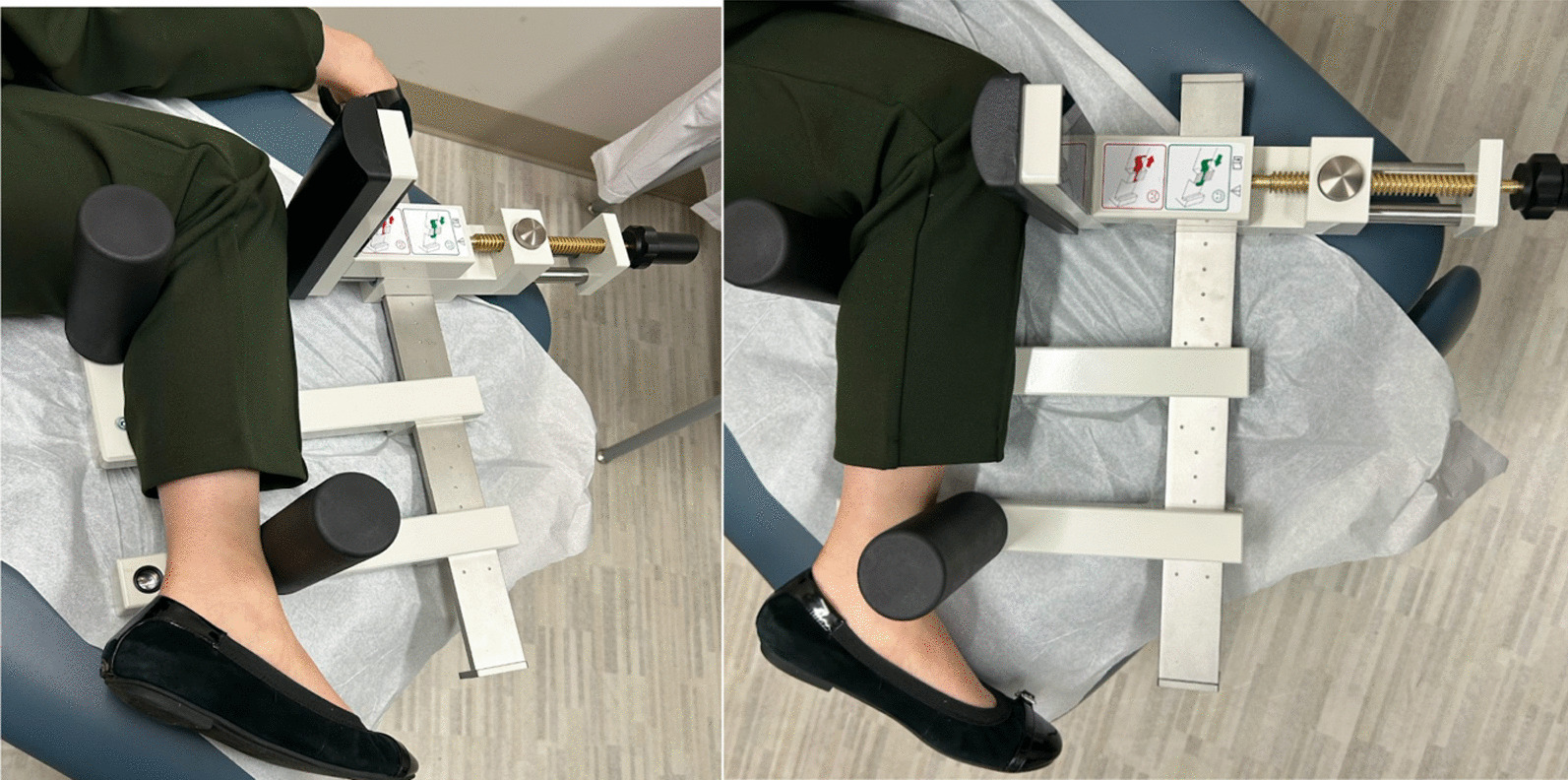
Telos stress device applying anterior force on the tibia through the knee

Following institutional review board approval, 10 patients who previously underwent TKA were prospectively enrolled for stress radiographic evaluation. Included patients were required to be at least 12 months from their arthroplasty procedure. Exclusion criteria included patients with more recent surgery as well as patients with hinged prostheses. Patients were enrolled consecutively in a single clinic to minimize selection bias. The study cohort included 10 patients (seven female, three male) with mean age 72 (range 55–82) years and an average duration from surgery of 36 (range 12–96) months. There were six left TKAs and four right TKAs. Liners included seven ultracongruent, one cruciate retaining, one posterior stabilized, and one varus–valgus constrained, which was included given a lack of constraint on anterior drawer testing. Enrolled patients underwent three calibrated lateral radiographs: at rest, Telos stress anterior drawer, and manual stress anterior drawer. Telos stress radiographs were performed at 150N per the manufacturer’s recommendation (Fig. 1). Manual stress was performed by a single provider performing an anterior drawer test until a stable endpoint was reached. Both tests were performed in the lateral position for radiographic evaluation.

**Fig. 2:**
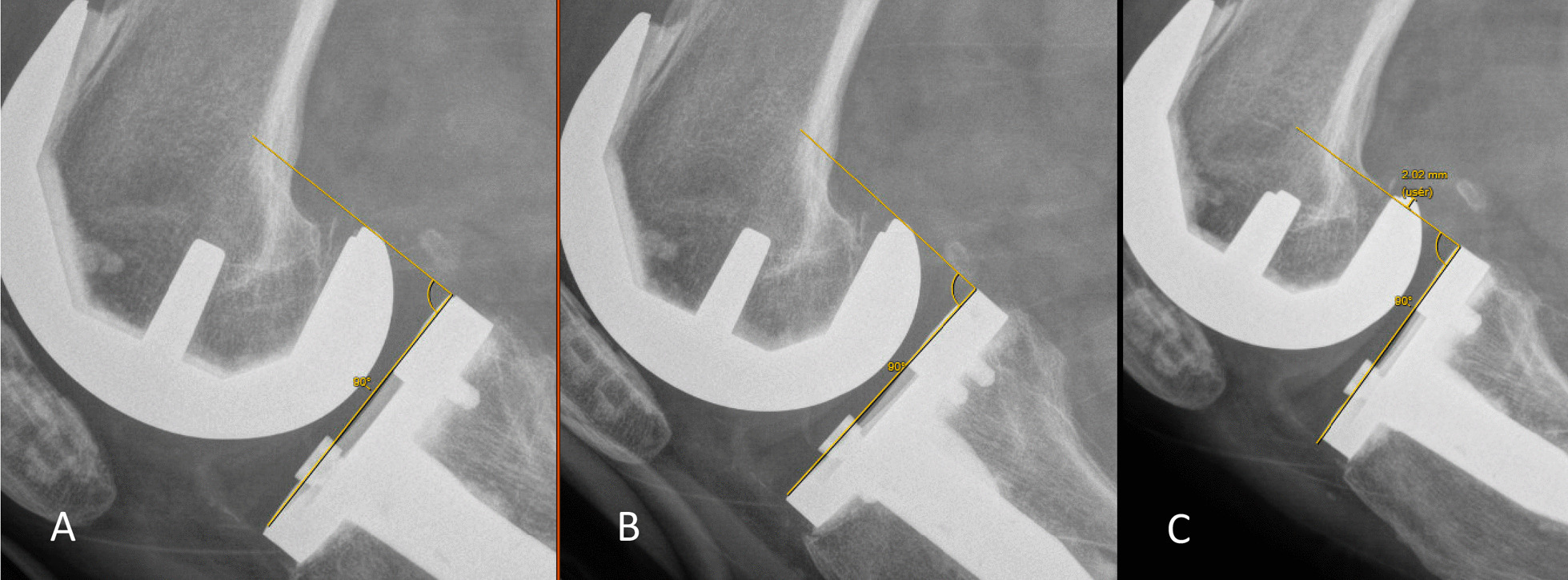
Anterior Tibial Translation **A** at rest **B** Telos stress, and **C** manual stress

Following radiographic evaluation, two raters measured the tibia position relative to the femur on each radiograph using the methods described by Seon et al. [10] and Moser et al. [12] at two different time-points separated by greater than one month. The differences in tibial position were calculated in millimeters with both Telos stress and manual stress relative to the position at rest (Fig. 2). These results were compared with Student's t test. Raters were blinded to each-others measures as well as to prior measurements performed. Measurements of translation < 1 mm were considered to be within the margin of error and were considered to have no translation. Intraclass correlation coefficients were utilized to evaluate intra- and inter-rater reliability, with values < 0.5 indicating poor reliability, 0.5–0.75 moderate reliability, 0.75–0.90 good reliability, and > 0.9 excellent reliability. *p* value < 0.05 was considered statistically significant.

## Results

The Telos stress device provided significantly less anterior translation than manual stress (mean: − 0.3 mm vs. 3.9 mm; *p* < 0.01). Two patients reported pain with use of the stress device compared to no discomfort with manual stress. One patient had no translation with either Telos or manual stress. This patient had a Zimmer Natural Knee II (Zimmer Biomet, Warsaw, IN) with ultracongruent polyethylene liner. Overall inter-observer reliability of measurements was good for telos stress (ICC = 0.86) and excellent for manual stress (ICC = 0.94). Eighty-five percent of all translation measurements were within 1 mm between observers. Intra-observer reliability of measurements was good to excellent for both Telos stress and manual stress (Table 1).

**Table 1:** Intra-observer Reliability

	Telos stress ICC	Manual stress ICC
*Intra-observer reliability of measurements*		
Observer A	0.81; *p* < 0.01	0.97; *p* < 0.01
Observer B	0.99; *p* < 0.01	0.98; *p* < 0.01

*ICC* intraclass correlation coefficient

## Discussion

While other sub-specialties of orthopedics have incorporated stress radiographs into common practice [13–15], adult reconstruction has infrequently utilized this diagnostic modality. Flexion instability remains one of the most common causes of revision for TKA patients, and may be a significant contributor to patient dissatisfaction; however, diagnostic evaluation and indications for surgical intervention remain highly subjective and surgeon dependent [1, 4–6, 16]. As a result, outcomes for revision TKA for flexion instability have been inconsistent in the literature [7, 8]. The current study found that a commercially available product did not adequately reproduce tibial translation when compared to manual stress. However, radiographic translation demonstrated good to excellent inter- and intra-observer reliability, indicating that stress radiographs may be reproducibly interpreted.

Current radiographic assessment of TKAs for flexion instability includes evaluation for excessive posterior slope and reduced posterior condylar offset on lateral X-rays as well as joint line elevation on AP radiographs [4, 5]. Unfortunately, no objective cutoff values or quantitative measures for these parameters have been identified. The existing literature indicates that A–P translation on physical examination > 10 mm should be considered marked translation, and there is suggestion that correction to < 5 mm translation is more appropriate for improved clinical outcomes [3, 4, 6]. However, A–P translation based on physical examination is inherently subjective, and no current literature to our knowledge utilizes objective quantitative measures to identify these patients. Importantly, prior authors have highlighted the subjective nature of knee laxity testing in TKA, with poor inter-rater reliability using clinical testing alone, discouraging its use in isolation [17].

It has previously been noted by Stambough et al. [4] that stress radiographs could be important in the diagnostic evaluation of flexion instability. However, no prior study has demonstrated that these radiographs can be reliably interpreted or correlated to outcomes after revision surgery. Prior to determining if stress radiographs correlate to patient outcomes, it is first prudent to determine if these radiographs can be reliably and reproducibly interpreted across providers. The current study is an important first step in creating objective criteria to identify flexion instability.

Seon et al. [10] utilized the Telos device for anterior as well as posterior drawer stress radiographs, and showed an association between postoperative range of motion and total anterior–posterior laxity. However, the measurement itself was not validated, and it was not utilized to determine patient satisfaction, but rather early range of motion. While these authors did find tibial translation with the commercially available device, they compared anterior to posterior drawer rather than anterior drawer to rest, which was performed in the current study. Other authors have utilized the stress device in a native knee for posterior drawer testing, and found high reproducibility for quantification of posterior instability [11]. However, it is unclear whether or not similar results can be seen in patients after TKA. The current study was unable to demonstrate successful tibial translation in patients after TKA.

There are several limitations to this study worth noting. First, flexion instability is a dynamic process which involves both anterior and posterior tibial translation throughout functional motion. The current study evaluated only anterior tibial translation at 90° of flexion in a static position. This was performed for two reasons: first, it is important to validate the radiographic measurements rather than correlate to patient outcome at this stage, and second, dynamic evaluation of translation with functional X-rays or 3-D imaging is unlikely to be widely utilized outside of tertiary academic centers. Additionally, this study was limited in that a single provider performed the stress radiographs, and it remains unclear if stress radiographs performed by different providers would have reproducible findings. Lastly, this study did not attempt to identify patients with flexion instability for inclusion. This was done intentionally, as the purpose of the current study was only to attempt to validate the radiographic measurement.

The current study was unable to demonstrate added value to a commercially available stress device for evaluation of flexion instability. Tibial translation with a stress device was significantly less than manual stress performed by a provider on physical examination, which is likely due to method of force application, rather than amount of force applied. However, the inter- and intra-observer reliability of radiographic measurements of stress radiographs for flexion instability was high. This is important to consider as future studies evaluating flexion instability can utilize manually performed stress radiographs, and tibial translation can be reproducibly measured. With this, adult reconstruction may be able to utilize stress radiographs to quantitatively identify patients with flexion instability, and correlate changes to their outcomes following revision surgery.

## Data Availability

Datasets are only accessible by the investigators and are housed in a secure network behind the institutional firewall.
